# Congenital heart disease: the children will become elderly

**DOI:** 10.18632/aging.101800

**Published:** 2019-01-28

**Authors:** Zacharias Mandalenakis, Kristofer Skoglund, Mikael Dellborg

**Affiliations:** 1 Department of Molecular and Clinical Medicine, Sahlgrenska University Hospital/Östra, Gothenburg, Sweden

**Keywords:** aging, congenital heart disease

Congenital heart disease is the most common congenital malformation occurring in just over 1% of live births. With the advances in noninvasive diagnostics, interventional catheterization, congenital heart surgery, cardiopulmonary bypass and postoperative care a dramatic change in the prognosis for children born with congenital heart disease has been observed the last decades [1,2]. Today more than 95% of children born with congenital heart disease reach adult age. While the relative risk of mortality during infant life remains substantially higher than for healthy persons of similar age it is never the less in absolute terms the risk is low. This will rapidly increase the number of, not only young adults, but also senior adults and elderly with congenital heart disease.

Patients with congenital cardiac malformations can be divided into those who do not need surgery (roughly half), because their condition will either be of none or very small significance (such as small ventricular septal defect [VSD], small atrial septal defect [ASD]) or because it will heal with time (such as spontaneous closure of VSD, ASD or ductus arteriosus). Those who have undergone an intervention by catheter and/or surgery and who which can either be considered “cured”, such as patients with persistent ductus arteriosus, VSD and small ASD treated with surgery or catheter. These patients are in general not followed up after childhood and will most likely have no late consequences of their condition. However, a large portion of patients, operated on in childhood may be considered as palliated. They will thus be subject to further medical, surgical and catheter-based interventions. Almost six out of 1000 adults will have a congenital condition that merits life-long follow-up [3].

The risk for the elderly patients with a palliated or un-operated congenital heart condition to develop atrial fibrillation is very high [4]. The same is true for ischemic stroke [5] and the relationship between atrial fibrillation and stroke certainly exists to a degree that earlier and more aggressive anticoagulation treatment to patient with congenital heart disease at least may be considered. However also arterial malformations may be more common in certain patients with congenital heart conditions, in particular patients with coarctation of the aorta where hemorrhagic stroke and subarachnoid bleedings are significantly overrepresented as compared to healthy adults of the same age [6]. Not surprisingly heart failure is also more common in the adult congenital heart patients, occurring at a higher rate and also at a much earlier age than heart failure in general. Less expected is the observed increased risk of coronary artery disease we, and others have found in young adults with congenital heart disease [7].

As for many persons and patients with other conditions, lifestyle has an important impact on the cardiovascular health also for patients with congenital heart disease. In general, the degree of physical activity tends to be lower for adult with congenital heart disease, possibly because of hesitation from patients themselves, from parents and teachers during childhood restricting physical activity. Many patients with congenital heart conditions are performing less well on a treadmill and this is to a significant extent, caused by lack of training, lack of muscular strength [8].

**Figure 1:**
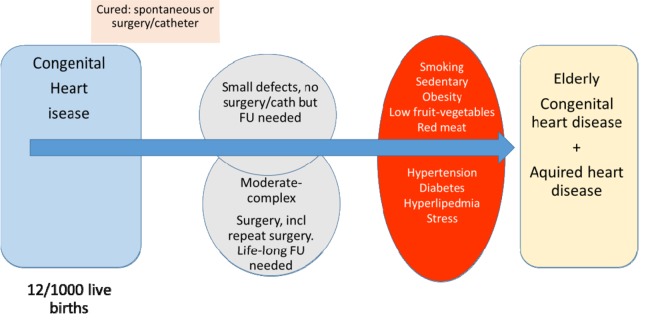
Congenital, acquired factors and lifestyle relevant for elderly with congenital heart disease.

The dramatic improvements in medical care for patients with congenital heart conditions have resulted in a dramatic increase in survival. At the same time the physicians caring for elderly patients will see increasing numbers of patients with congenital heart disease who survive until old age. They will bring with them an increased risk of cardiovascular morbidity, such as atrial fibrillations, stroke, intracerebral hemorrhage, acute myocardial infarction and heart failure. On top of these inherited risks, patients with adult congenital heart disease will also be subject to the negative effects of an unhealthy lifestyle. Regardless of age these patients should be considered, and are at a higher than average risk for mortality and morbidity. While we do not yet specifically know when or where and when we should start preventive medical treatment such as anticoagulation or cholesterol-lowering treatment, meticulous care to medical treatment and to lifestyle factors is warranted, as illustrated in Figure 1. The care of elderly patients with congenital heart conditions necessitates a very close and open cooperation between the specialized congenital care cardiologist and the general practitioner and/or internist.
